# Identification of Stromal Cells in Spleen Which Support Myelopoiesis

**DOI:** 10.3389/fcell.2019.00001

**Published:** 2019-01-24

**Authors:** Hong Kiat Lim, Helen C. O’Neill

**Affiliations:** ^1^Clem Jones Centre for Regenerative Medicine, Faculty of Health Sciences and Medicine, Bond University, Gold Coast, QLD, Australia; ^2^Research School of Biology, Australian National University, Canberra, ACT, Australia

**Keywords:** hematopoiesis, stroma, spleen, myelopoiesis, microenvironment

## Abstract

Stromal cells in spleen organize tissue into red pulp, white pulp and marginal zone, and also interact with hematopoietic cells to regulate immune responses. This study has used phenotypic information of a previously described spleen stromal cell line called 5G3, which supports restricted hematopoiesis *in vitro*, to identify an equivalent stromal cell subset *in vivo* and to test its capacity to support hematopoiesis. Using stromal cell fractionation, phenotypic analysis, as well as cell growth and hematopoietic support assays, the Sca-1^+^gp38^+^Thy1.2^+^CD29^+^CD51^+^ fraction of spleen stroma has been identified as an equivalent stromal subset resembling the 5G3 cell counterpart. While heterogeneity may still exist within that subset, it has been shown to have superior hematopoietic support capacity compared with the 5G3 cell line, and all other spleen stromal cell fractions tested.

## Introduction

Stromal cells of mesenchymal lineage represent a supporting cellular network forming the basement layer in tissue and contributing to organ architecture and function (Mueller and Germain, 2009; Roozendaal and Mebius, 2011). In the case of spleen, distinct stromal cells are identifiable in the regions of red pulp, white pulp and marginal zone. So far, several distinct mesenchymal stromal cell types have been described in spleen. Splenic fibroblastic reticular cells assist in regulating immune responses through secretion of chemokines like CCL19 and CCL21 (Luther et al., 2000). Others include gp38^+^ fibroblastic reticular cells, MadCAM1^+^ marginal reticular cells, CD35^+^ follicular dendritic cells, CD105^+^ red pulp fibroblasts and CD31^+^ vascular endothelial cells (Mueller and Germain, 2009; den Haan et al., 2012).

Many studies have investigated the function of splenic stroma in immune regulation (Malhotra et al., 2013), but limited studies have addressed their hematopoietic support capacity. The perception in the field is that extramedullary hematopoiesis occurs in spleen with stress or disease. However, questions remain about the role of spleen in steady-state hematopoiesis (Kim, 2010; Johns and Christopher, 2012). A role for spleen in hematopoiesis has been clearly defined in mice, and several studies now indicate the presence of hematopoietic stem cells (HSC) in spleen of several species including humans (Dor et al., 2006; Tan and O’Neill, 2010; Inra et al., 2015). Definition of a role for spleen in hematopoiesis would offer tremendous clinical potential, and the substantial knowledge of bone marrow hematopoiesis could easily be directed to a study of hematopoiesis in spleen. Furthermore, there has been no in-depth analysis of splenic stroma which could be candidate elements for HSC niches.

Previously, we described a cloned stromal cell line called 5G3 which is unique in its ability to support *in vitro* hematopoiesis. When 5G3 stroma was overlaid with bone marrow progenitors, transient production of myeloid and conventional dendritic-like cells (cDC) was reported, as well as the continuous production of a specific dendritic-like cell called ‘L-DC’ (Periasamy et al., 2009; Petvises and O’Neill, 2014a,b). The cDC-like cells were recently identified as regulatory DC (Petvises et al., 2018). Several studies also identified the maintenance of progenitors within co-cultures (Tsuchiyama et al., 1995; Corselli et al., 2013; Petvises and O’Neill, 2014a), and the ability to achieve L-DC production through overlay of HSC or multipotential progenitors (MPP) above stroma (Hinton et al., 2011; Petvises and O’Neill, 2014b). Longterm stromal cocultures maintain HSPC and this has been demonstrated through *in vivo* reconstitution assays (O’Neill et al., 2014). The 5G3 splenic stromal line expresses mesenchymal markers like CD140a, CD51, CD29, gp38, Thy1, Sca-1, and CD105 (Lim et al., 2018). Attempts have been made here to isolate an *in vivo* equivalent stromal cell subset to 5G3 and to compare its hematopoietic support capacity with other stromal fractions. This study uses marker analysis to define stromal subsets in spleen and to assess their capacity for *in vitro* growth. It also identifies subsets which support hematopoiesis which could represent candidate niche elements for hematopoiesis in spleen. This *in vivo* study therefore provides physiological relevance to studies describing *in vitro* hematopoiesis.

## Materials and Methods

### Animals

Specific pathogen-free C57BL/6J (*H-2K^b^: CD45.2*) mice aged 6 days or 4–8 weeks were obtained from the John Curtin School of Medical Research (JCSMR: Canberra, ACT, Australia). Mice were housed and handled according to protocols approved by the Animal Experimentation Ethics Committee at the Australian National University (ANU: Canberra, ACT, Australia).

### Cell Cultures

The 5G3 stroma was derived from the STX3 splenic stromal line by single-cell cloning and freezing down (Despars and O’Neill, 2006a,b; Despars et al., 2008). Cells were cultured at 37°C in 5% CO_2_ in air with 95% humidity in supplemented Dulbecco’s modified Eagle’s medium (Sigma-Aldrich: Castle Hill, NSW, Australia) containing 10% fetal calf serum, 5 × 10^-4^ M 2-mercaptoethanol, 10 mM HEPES, 100 U/ml penicillin, 100 μg/ml streptomycin, 4 mg/L glucose, 6 mg/L folic acid, 36 mg/L L-asparagine, 116mg/l L-asparagine hydrochloric acid (sDMEM). Stromal cells were passaged twice by transferring scraped cells into new flasks before use in experiments.

### Fractionation of Bone Marrow Progenitors

Methods for isolation of bone marrow progenitors have been described (Periasamy et al., 2013; Petvises and O’Neill, 2014a). Bone marrow cells were flushed from femurs of C57BL/6J adult mice. Red blood cells were selectively lysed using lysis buffer. MACS^®^ magnetic bead technology (Miltenyi Biotec: Gladbach, Germany) was used to deplete lineage-specific hematopoietic cells. The process involved labeling cells with a cocktail of lineage-specific biotinylated antibodies with specificity for NK1.1, CD11b, CD11c, Gr-1, MHC-II, Ter119, CD3 and CD19, followed by anti-biotin microbeads, which were captured in LS or MS columns (Miltenyi Biotec). Lin^-^ bone marrow progenitors were eluted and collected.

### Dissociation of Splenic Stromal Cells

Murine adult or 6 day old spleens were dissociated by pressing between microscope slides before filtering through a 70 μm strainer. The stromal fraction was collected in a tube containing 2 ml collagenase IV extraction buffer [(2% fetal calf serum, 1 mg/ml collagenase IV (Sigma-Aldrich) and 40 μg/ml DNase I (Sigma-Aldrich) in RPMI] and incubated for 20 min at 37°C with rotation. Two ml collagenase D extraction buffer [2% fetal calf serum, 1 mg/ml collagenase D (Roche Applied Science: North Ryde, NSW, Australia) and 40 μg/ml DNase I in RPMI] was added for another 20 min incubation at 37°C with rotation. An additional 2 ml of collagenase D extraction buffer was added and the cell suspension incubated for 20 min at 37°C with rotation. The activity of collagenase was halted by addition of 60 μL of 500 mM EDTA to give a final concentration of 5 mM. Digested spleens were washed twice with 5 ml sDMEM (300 g, 4°C, 5 min), and viable cell count determined using trypan blue staining. The cell pellet was resuspended in 1ml sDMEM for antibody staining, flow cytometric analysis and sorting.

### Antibody Staining and Flow Cytometric Analysis

The procedures used to stain cells and assess antibody binding have been described (Periasamy et al., 2009; Periasamy and O’Neill, 2013; Petvises and O’Neill, 2014b). Antibodies used were specific for CD11b (clone M1/70), CD11c (N418), NK1.1 (PK136), Gr-1 (RM6-8C5), Ter119 (TER119), CD3 (145-2C11), CD19 (6D5), c-Kit (2B8), FLT3 (A2F10), CD150 (TC15-12F12.2), MHC-II (AF6-120.1), F4/80 (BM8), CD45.2 (104), CD29 (HMβ1-1), CD51 (RMV-7), CD54 (YN1/1.7.4), CD31 (390), gp38 (8.1.1), CD105 (MJ7/18), Thy1.2 (30-H12), VCAM1 (429), CD140a (APA5), CD146 (ME-9F1), Sca-1 (D7), ER-TR7 (Sc-73355), MAdCAM1 (MECA-367). Conjugates included streptavidin-Alexa780, streptavidin-APC-Cy7, streptavidin-PE, and streptavidin-FITC. Antibodies and conjugates were purchased from Biolegend (San Diego, CA, United States) or eBiosciences (Parkville, Victoria, Australia). Fluorescence minus one (FMO) controls or isotype controls were used to set gates to delineate specific antibody binding. Discrimination of dead cells was carried out following staining with 1 μg/ml propidium iodide (PI). Flow cytometric analysis and sorting utilized either a FACSDiva or a LSRII flow cytometer (Becton Dickinson: Franklin Lakes, NJ, United States). Post-acquisition analysis was carried out using FlowJo software (Tree Star: Ashland, OR, United States). For isolation of stromal subsets, cells were sorted into FACS buffer without added sodium azide, washed twice with phosphate buffered saline (Sigma-Aldrich), and plated for *in vitro* growth analysis. Sorted cells were re-analyzed flow cytometrically to ensure that purity of the sort was >99%. For sorting HSC, Lin^-^ bone marrow progenitors were prepared and stained with fluorochrome-conjugated antibodies to lineage markers, as well as specific markers. The longterm (LT)-HSC subset was isolated as Lin^-^Sca-1^+^c-Kit^+^Flt3^-^CD150^+^ cells (Kiel et al., 2005).

### Culture of Stromal Fractions

Stromal cells sorted by flow cytometry were cultured (5% CO_2_ in air with 95% humidity at 37°C) in a 6-well plate containing sDMEM for 28 days or until about 90% confluent. Cells were passaged from 6-well plates into a 25 cm^2^ flask and maintained until 90% confluency was obtained. Cells underwent a second passage from 25 cm^2^ into 75 cm^2^ flasks. Cells in the 75 cm^2^ flasks were either analyzed for cell surface marker expression using flow cytometry, or tested for hematopoietic support capacity in co-culture assays.

### Stromal Co-cultures

In order to assess hematopoietic support capacity of stroma, Lin^-^ bone marrow cells were prepared as above and overlaid at 1–5 × 10^4^ cells/ml in 20 ml sDMEM above stromal monolayers of 80–90% confluency. In some experiments, HSC were overlaid at 1–5 × 10^2^ cells/ml in 5 ml sDMEM above stroma. Co-cultures were kept at 37°C, 5% CO_2_ in air and 97% humidity. Production of cells in co-cultures was monitored over a period of 4–6 weeks using flow cytometry and light microscopy. Since co-cultures established at different times varied in cell yield over the course of culture, each test of hematopoietic support capacity included 5G3 stroma as a control. At 7-day intervals, non-adherent cells were collected by aspiration and replacement of medium. Trypan blue exclusion was used to determine cell yield. Cells were then resuspended in FACS buffer for flow cytometry, in order to detect cell surface marker expression and to define and quantitate subsets.

### Gene Expression Analysis

Gene expression was measured by quantitative real time polymerase chain reaction (qRT-PCR). Total RNA was isolated from stromal cell lines using the RNeasy mini kit and the manufacturer’s protocol (Qiagen, SABiosciences: Valencia, CA, United States). Genomic DNA elimination mix was added to 400–600 μg of RNA followed by incubation for 5 min at 42°C to purify RNA. Following this, Buffer BC3, Control P2, Reverse Transcriptase mix and RNase-free water were added in ratios of 4:1:2:3 for preparation of cDNA. Denaturation proceeded for 15 min at 42°C, then for 5 min at 95°C to convert RNA into cDNA. Equal volumes of cDNA and primer were mixed. Primers were purchased from SABioscience (Frederick, MD, United States: *Scf*: PPM02983C; *Cxcl12*: PPM02965E; *Actb*: PPM02945A). The cDNA/primer mix was then added to the RT^2^ SYBR Green Mastermix and RNase-free water in a ratio of 1:6.25:5.25, respectively. Samples were loaded on to a LightCycler 480 (Roche: Penzberg, BAV, Germany) with cycling conditions: 1 cycle for 10 min at 95°C to activate DNA Taq Polymerase, followed by 45 cycles of 15 s at 95°C for extension, and then 1 min at 60°C for fluorescence data collection. Roche LightCycler 480 software v.11.2.9.11 was used to analyze qRT-PCR data. Derivation of crossing point (*C*_p_) was carried out using the absolute quantification (2nd derivative max) method at high confidence. *C*_p_ is defined as the point at which the maximal increase in fluorescence occurs within the log-linear phase. The *C*_p_ value is also referred to as threshold cycle (*C*_t_). Δ*C*_t_ = *C*_t_ (*G*_i_) -*C*_t_(*G*_r_), where *G*_i_ refers to gene of interest and *G*_r_ refers to reference gene. Fold change relative to β-*actin* was expressed as 2^-ΔCt^ (gene of interest)/2^-ΔCt^ (β-*actin*).

### Microscopy

Cell morphology was observed and photographed using an EVOS^®^ FL digital fluorescence microscope (Electron Microscope Sciences: Hatfield, PA, United States), equipped with a Sony^®^ ICX445 CCD camera (Sony: Minato, TKY, JP).

### Statistical Analysis

Data are presented as mean ± standard error (SE) for sample size *n*. The Student’s *t*-test was used to assess significance (*p* ≤ 0.05).

## Results

### Composition of Splenic Stroma

In order to investigate the stromal cell composition of murine spleens, collagenase-dissociated stromal cells were fractionated using flow cytometry to enrich or deplete subsets expressing a particular marker(s). Previously 6 day old spleens were found to give optimal production of longterm stroma-dependent cultures supporting hematopoiesis, although other ages could be used but with less effectiveness. For this reason, 6 day old mice were used for characterization of splenic stromal subsets. The initial choice of markers was based on the phenotype of the 5G3 line, as well as knowledge of mesenchymal cells. A high proportion of splenic stromal cells (91.70 ± 4.05%) was found to express CD29 (integrin beta-1) (Table 1). In contrast, markers like Sca-1, CD31, gp38, CD140a, CD51, ER-TR7, Thy1.2, CD146, MadCAM1 and VCAM1 were expressed by minority populations of stroma (1–3%) (Table 1). CD105^+^ cells represented ∼13% of stromal cells in spleen, with VCAM1^+^ ∼7%. Splenic stromal cell characterization was therefore challenging, requiring analysis and isolation of rare stromal cells expressing markers of interest.

**Table 1:** Proportion of cells in CD45^-^ stromal fractions.

Spleen fraction^a^	% cells^b^
CD29^+^	91.70 ± 2.70
Sca-1^+^	1.57 ± 0.50
CD31^+^	1.02 ± 0.23
gp38^+^	0.99 ± 0.32
CD105^+^	13.07 ± 1.26
CD140a^+^	1.76 ± 0.67
CD51^+^	1.64 ± 0.73
ER-TR7^+^	2.12 ± 0.67
Thy1.2^+^	0.32 ± 0.11
CD146^+^	1.33 ± 0.36
MAdCAM1^+^	0.35 ± 0.06
VCAM1^+^	6.82 ± 3.60

^a^*Stromal cells were isolated from neonatal murine spleen using collagenase treatment. Cells were labeled with fluorochrome-conjugated antibodies and flow cytometry used to separate marker-positive and marker-negative fractions for quantification.*^b^*Data represent mean ± SE (n = 3)*.

In developing a strategy for detection of stromal subsets which support hematopoiesis, we were cognizant of the fact that the 5G3 phenotype may not accurately reflect the stromal cell from which 5G3 derived. We therefore performed a systematic study of marker defined stromal fractions to eliminate subsets which did not grow, and which did not support hematopoiesis. An important criterion was the capacity of the stromal fraction to grow to confluency within 28 days in order to test hematopoietic support capacity. This approach precluded multicolor sorting yielding very small numbers of cells which could not form a monolayer *in vitro*.

In a first experiment, marker identity of fractions which grew *in vitro* was determined by comparing growth capacity of subsets enriched or depleted of cells expressing a given marker. Markers were considered unimportant when the same growth capacity was achieved for depleted and enriched fractions (either did or did not grow), and where depletion and not enrichment gave a growing population. Depletion of stroma expressing the endothelial markers CD31 and VCAM1 gave a stromal population which grew readily, so these markers were removed from consideration (Table 2). Stromal cells enriched or depleted for CD146 and MadCAM, known markers of niche elements in bone marrow, did not grow despite multiple replicated experiments (Table 2). This could be due to inability to isolate enough cells, or their inherent inability to form a monolayer, so these markers were eliminated from further consideration. The more common stromal cell markers Sca-1, CD51 and ERTR7, were not deterministic of growth since both depleted and enriched subsets grew (Table 2). This initial screen led to the identification of CD29, gp38, CD105, CD140a and Thy1 as deterministic markers. Only fractions enriched for, but not depleted of CD29, gp38, CD105, Thy1.2 and CD140a, could reach confluent growth by 28 days (Table 3). These mesenchymal markers would appear to identify stromal cells that can replicate in culture. Subsets expressing these markers were therefore tested for hematopoietic support capacity.

**Table 2:** Phenotype of stromal fractions after culture.

Cell fractions^a,b^	Growth^c^	Phenotype of 28 day stroma^d^						
		Sca1	gp38	CD51	CD105	ERTR7	CD140a	Thy1.2
**Endothelial cell markers**								
CD31^+^ (*n* = 1)	-							
CD31^-^ (*n* = 1)	^∗∗^	++	+	+++	-	-	+	+
VCAM1^+^ (*n* = 1)	-							
VCAM1^-^ (*n* = 1)	^∗^	+++	+++	+++	+++	-	-	+++
**PVRC markers**								
CD146^+^ (*n* = 4)	-							
CD146^-^ (*n* = 4)	-							
MadCAM1^+^ (*n* = 3)	-							
MAdCAM1^-^ (*n* = 3)	-							
**Common stromal markers**								
Sca-1^+^ (*n* = 3)	^∗^	+++	++	+++	+	-	-	+++
Sca-1^-^ (*n* = 3)	^∗^	+++	++	+++	+	-	-	++
CD51^+^ (*n* = 1)	^∗^	+++	+++	+++	+	-	+	+++
CD51^-^ (*n* = 1)	^∗^	+++	+++	+++	++	-	-	+++
ER-TR7^+^ (*n* = 2)	^∗^	+++	++	+++	-	-	-	+++
ER-TR7^-^ (*n* = 2)	^∗^	+++	+++	++	+++	-	-	-

^a^*Stromal cells were isolated from murine spleen using collagenase treatment and stained with antibodies specific for CD45.2 to gate out hematopoietic cells. They were then stained for known markers of endothelial cells (CD31 and VCAM), perivascular reticular cells (PVRC) (CD146, MadCAM1) or common stromal cell markers (Sca-1, CD51 and ER-TR7) to gate distinct fractions. Where indicated, replicate independent sorting experiments were conducted (n). Sorted subsets were cultured in sDMEM for 28 days.*^b^*1–3 × 10^*4*^ cells were plated, except for the MadCAM1^+^ subset where ∼3.4 × 10^*3*^ cells were available for plating.*^c^*Cell growth: confluent by 10 days (^∗∗^), confluent by 28 days (^∗^), no confluence (-).*^d^*Cells were trypsinised and stained with antibodies to determine phenotype. +++, >80% cells expressing; ++, 50–80%; +, 10–50%; -, <10%.*

**Table 3:** Further phenotyping of stromal fractions after culture.

Cell fractions^a,b^	Growth^c^	Phenotype of 28 day stroma^d^						
		Sca1	gp38	CD51	CD105	ERTR7	CD140a	Thy1.2
CD29^+^ (*n* = 2)	^∗∗^	+++	+++	+++	+++	-	+	+++
CD29^-^ (*n* = 2)	X							
gp38^+^ (*n* = 5)	^∗^	+++	+++	++	++	-	++	++
gp38^-^ (*n* = 5)	-							
CD105^+^ (*n* = 5)	^∗^	+++	+++	++	+++	-	+	++
CD105^-^ (*n* = 5)	-							
CD140a^+^ (*n* = 5)	^∗^	++	+++	++	-	-	-	++
CD140a^-^ (*n* = 5)	-							
Thy1.2^+^ (*n* = 3)	^∗^	++	+	+	+	-	++	+++
Thy1.2^-^ (*n* = 3)	X							

^a^*Stromal cells were isolated from murine spleen using collagenase treatment and stained with antibodies specific for CD45.2 to gate out hematopoietic cells. They were then stained for either CD29, gp38, CD105, CD140 or Thy1.2 which distinguished subsets which grew well in vitro. Independent, replicated sorting experiments were conducted to confirm results (n). Sorted cell subsets were cultured in sDMEM for 28 days.*^b^*1–3 × 10^*4*^ cells were plated, except for the more prevalent CD29^+^ cells, where 1.6 × 10^*6*^ cells were plated.*^c^*Cell growth: confluent by 10 days (^∗∗^), confluent by 28 days (^∗^), no confluence (-), and no growth (X).*^d^*Cells were trypsinised and stained with antibodies to determine phenotype. +++, >80% cells expressing; ++, 50–80%; +, 10–50%; -, <10%.*

Cells which grew out of 28-day cultures were also mesenchymal in terms of phenotype (Tables 2, 3). For all stromal fractions tested, cells which grew expressed Sca-1 (50–80% of cells), with a majority of cells also expressing gp38, CD51 and Thy1.2 (50–80%). The expression of CD105 was variable, with some cultures showing no expression. CD140a expression was also weak and variable with between 0 and 50% of cells expressing this marker. None of the cultures showed expression of the fibroblastic marker ER-TR7. These data need to be interpreted with caution since *in vitro* growth can moderate or enhance expression of markers. In sum, a small number of lines of distinct mesenchymal type can be cultured, showing variability in expression of CD105 and CD140a.

### Microscopic Examination of Cultured Stromal Fractions

**Figure 1:**
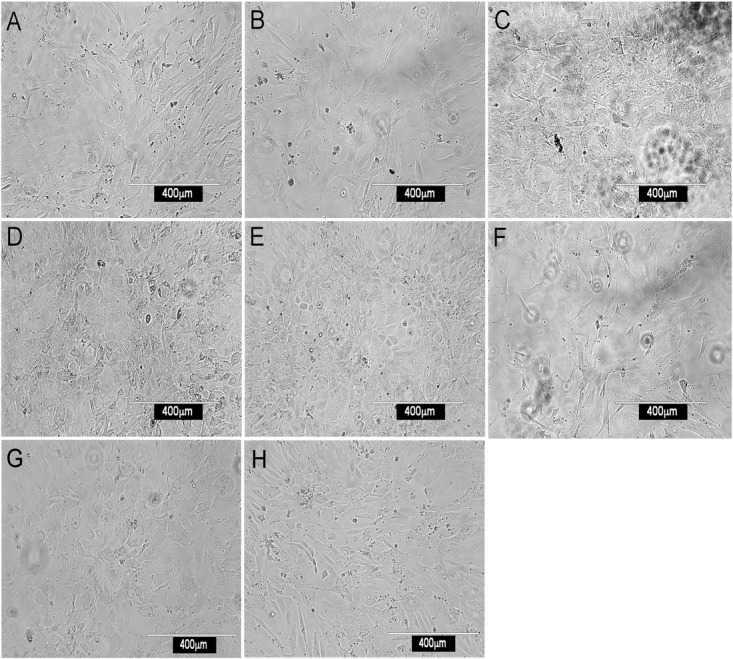
Morphology of splenic stromal fractions. Stromal cells were isolated from murine spleen using collagenase treatment, stained with antibody and sorted flow cytometrically. Cells were cultured and photographed after 28 days using a camera attached to an inverted phase microscope. Stromal fractions sorted on the basis of a single marker were tested for *in vitro* growth. **(A)** CD29^+^, **(B)** Sca-1^+^, **(C)** CD140a^+^, **(D)** CD105^+^, **(E)** gp38^+^, **(F)** ERTR7^+^, **(G)** CD51^+^ and **(H)** Thy1.2^+^.

Most stromal monolayers were heterogeneous showing a mix of spindle-shaped fibroblastic and cuboidal cells. The extent of confluency achieved for different fractions varied even after 28 days of culture. Enrichment of CD29^+^, Sca1^+^, CD140a^+^, CD105^+^, gp38^+^, CD51^+^ and to a lesser extent Thy1.2^+^ cells, gave confluent, extensive monolayers comprising mainly spindle-shaped cells, reflective of mesenchymal stroma (Figure 1). Enrichment for CD29^+^ cells gave a very mixed population of cuboidal and spindle-shaped cells, consistent with CD29 as a marker of ∼90% of stromal cells in spleen (Table 1). The extensive heterogeneity within the CD29^+^ fraction was revealed by culturing CD29-expressing cells as three independent subcultures (A, B, and C) for 28 days. Stromal cells in each of A, B, and C subcultures showed very different growth rates and morphologies. Sub-culture A reflected spindle-shaped cells, sub-culture B sparse cuboidal cells, and sub-culture C was a confluent mixture of cell types (data not shown). The ER-TR7^+^ fibroblastic population grew rapidly yielding fibroblastic cells (Figure 1).

### Further Marker Characterization of Stroma

Since CD29^+^ cells represented ∼90% of stroma, subfractions were isolated on the basis of CD105, Sca-1 and gp38 expression, and compared for growth. In general, stromal fractions that expressed either CD29 or gp38 grew well (Supplementary Table S1). Again, the phenotype of stromal cells which grew out of 28 days cultures was consistently Sca-1^+^gp38^+^Thy1.2^+^CD29^+^CD51^+^, with variability in the expression of CD105 and CD140a (Supplementary Table S1).

**Figure 2:**
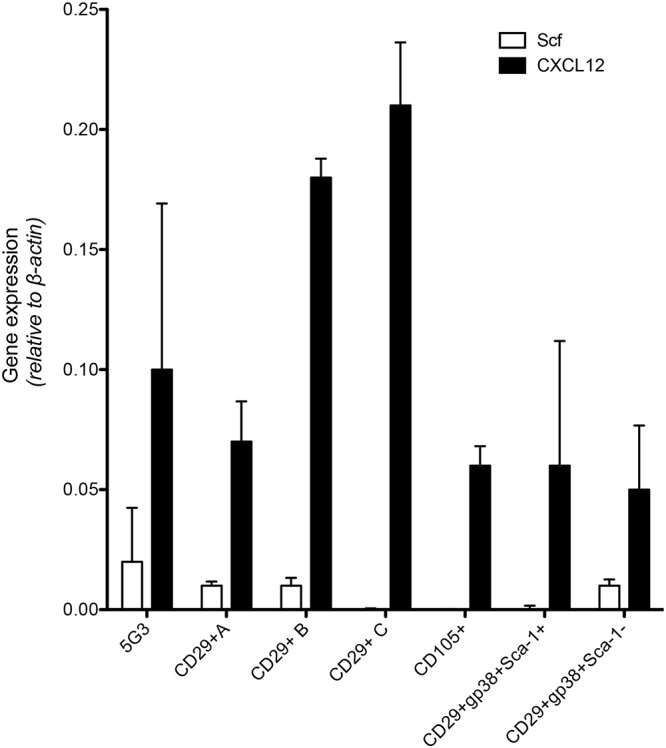
Expression of hematopoietic factors by spleen stromal fractions. Gene expression in 5G3 stroma was compared with several sorted stromal fractions of spleen using qRT-PCR. Data represent the mean ± SE of gene expression measured as fold change relative to the *Actb (b-actin)* housekeeping gene for four replicated samples. Expression of *Cxcl12* and *Scf* by all stromal fractions tested was not significantly different from 5G3 (*p* < 0.05).

As a prelude to assessment of potential of subsets to support hematopoiesis, qRT-PCR was used to measure expression of *Scf* and *Cxcl12* (Figure 2) which encode factors known to regulate hematopoiesis in bone marrow (Sugiyama et al., 2006). Recent work on spleen stroma has aligned *Cxcl12* expression with perivascular reticular cells, and *Scf* expression with both endothelial and perivascular reticular cells in the red pulp of murine spleen (Inra et al., 2015). The A, B, and C subcultures derived from an isolate of CD29^+^ stromal cells were compared with 5G3 stroma and with several sorted fractions of CD105^+^, CD29^+^gp38^+^Sca-1^-^ and CD29^+^gp38^+^Sca-1^+^ spleen stroma, to measure gene expression relative to β-*actin* (Figure 2). *Cxcl12* was highly expressed in all stroma, and each not significantly different from 5G3 at *p* = 0.05. *Scf* was more weakly expressed, not detectable in 3 subsets, and each not significantly different from 5G3 at *p* = 0.05 level. The dominant expression of *Cxcl12* and weak expression of *Scf* by stromal fractions is consistent with perivascular reticular cells amongst the spleen stromal fractions (Inra et al., 2015).

An explanation for the data achieved to date is that one or a small number of stromal subsets exist in spleen which are readily cultured *in vitro*. These are identifiable as a CD29^+^Sca-1^+^Thy1^+^CD51^+^gp38^+^ population, and contain perivascular reticular cells.

### Assessment of Hematopoietic Support Capacity of Stromal Fractions

**Figure 3:**
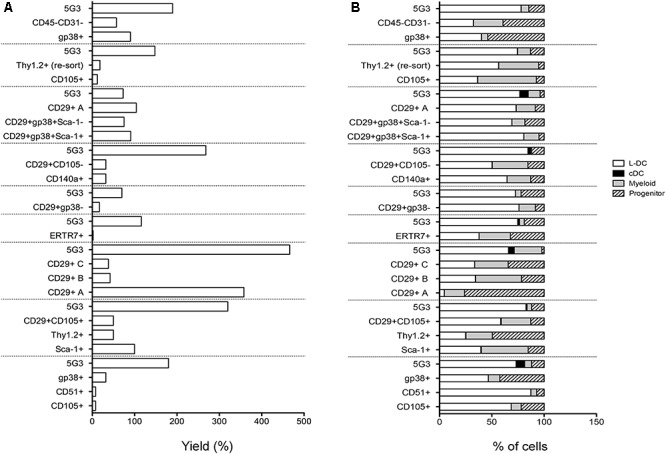
*In vitro* hematopoietic support capacity of stromal cell fractions. Stromal fractions were isolated from collagenase treated spleen stroma, sorted on the basis of marker expression and cultured for 28 days until confluent. Lin^-^ bone marrow cells was then overlaid above stroma at 1–5 × 10^4^ cells/ml in 20 ml. Non-adherent cells were collected after 21 days and stained with antibodies to CD11b, CD11c, MHC-II and F4/80 to delineate myeloid/dendritic cell production. Flow cytometric analysis was used to identify subsets of progenitors (CD11b^-^CD11c^-^), myeloid cells (CD11b^+^CD11c^-^F4/80^-^MHC-II^-^), L-DC (CD11b^+^CD11c^+^F4/80^+^MHC^-^II^-^), and cDC-like cells (CD11b^+/-^CD11c^+^F4/80^-^MHC^-^II^+^). Multiple individual self-controlled experiments were conducted, each comparing cell production by different fractions in comparison with 5G3. Data is shown for each of 9 experiments as: **(A)** % yield as number of cells produced relative to input cell number, and **(B)** proportional representation of subsets.

**Figure 4:**
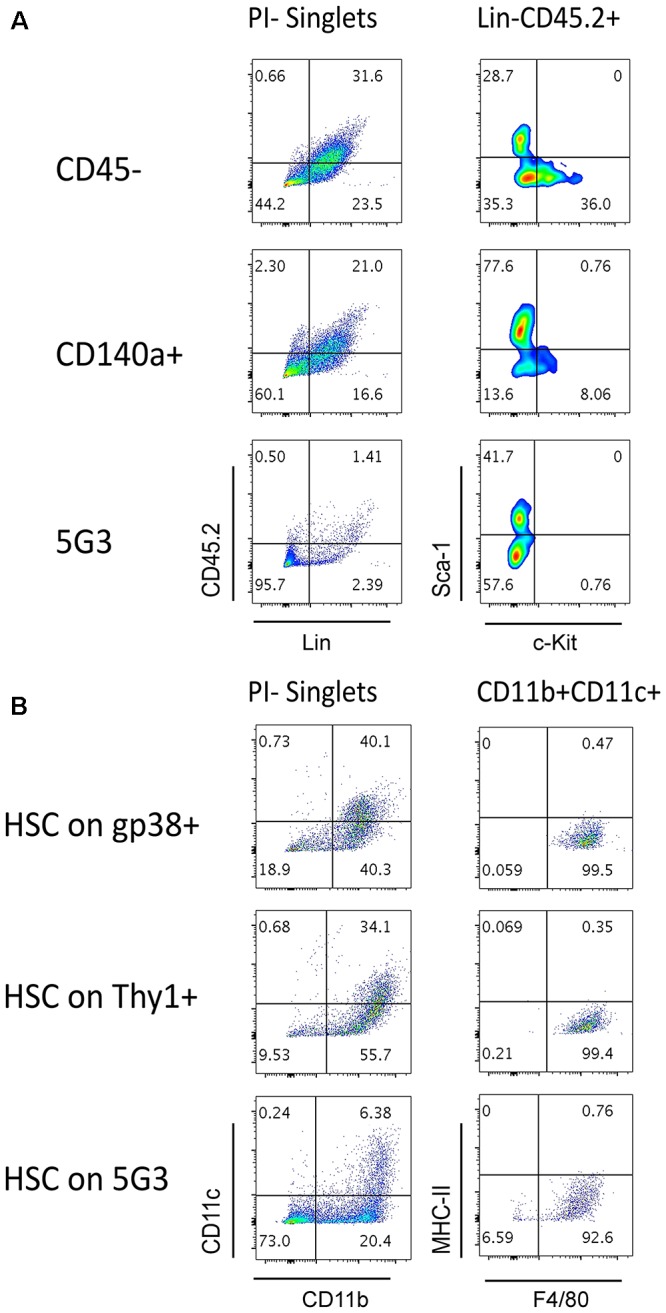
Splenic stroma supports hematopoietic progenitors. Stromal fractions expressing gp38, Thy1 or CD140 along with the CD45^-^ fraction were sorted and cultured for 28 days until confluent. 5G3 stroma was used as a control. Co-cultures were prepared as described in Figure 3 utilizing Lin^-^ bone marrow progenitors in **(A)** or sorted Lin^-^Sca-1^+^c-Kit^+^Flt3^-^CD150^+^ LT-HSC in **(B)**. Cultures were trypsinised at cell harvest to ensure isolation of any progenitors adherent to stroma before staining with antibodies to delineate cell subsets. Fluorescence minus one (FMO) controls were used to set gates. **(A)** Cells harvested at 35 days were stained for lineage (Lin) markers and CD45.2, as well as c-Kit and Sca-1, to identify hematopoietic progenitors. **(B)** Cells harvested at 28 days were stained for CD11b, CD11c, MHC-II, and F4/80 to delineate myeloid/dendritic cell production: myeloid cells (CD11b^+^CD11c^-^F4/80^-^MHC-II^-^), L-DC (CD11b^+^CD11c^+/-^F4/80^+^MHC^-^II^-^), regulatory DC (CD11b^+/-^CD11c^+^F4/80^-^MHC^-^II^+^) and progenitor-containing subset (CD11b^-^CD11c^-^).

To assess hematopoietic support capacity of stromal fractions, confluent monolayers were grown for 28 days and used to establish co-cultures by overlay of Lin^-^ bone marrow cells from CD45-allotype distinct mice. The production of progenitors, myeloid cells and dendritic cells was measured at 21 days through staining with a range of antibodies. The CD11b^-^CD11c^-^ subset was identified as ‘progenitors’ although this phenotype describes a heterogeneous population of progenitors/precursors. Myeloid cells were identified as CD11b^+^CD11c^-^F4/80^-^MHC-II^-^, L-DC as CD11b^+^CD11c^+^F4/80^+^MHC-II^-^ cells and regulatory DC cells as CD11b^+^CD11c^+^F4/80^+/-^MHC-II^+^. Initial studies involved stromal cell fractions separated by a single marker. Eleven independent co-culture experiments were performed to test various stromal fractions using 5G3 as a control. Nine representative experiments show % yield of cells relative to input cell number (Figure 3A). An example of the flow cytometric gates used to identify cell subsets produced in co-cultures is shown in Figure 4B and has also been publishedpreviously (Lim et al., 2018). Most stromal fractions were low cell producers compared with 5G3, including CD105^+^, CD51^+^, gp38^+^, Sca-1^+^, Thy1.2^+^, CD29^+^CD105^+^, CD29^+^B and CD29^+^C, ERTR7^+^, CD29^+^gp38^-^, CD140a^+^, CD29^+^CD105^-^ and CD45^-^CD31^-^ stroma. Several stroma gave yields equal to 5G3 including CD29^+^A, CD29^+^gp38^+^Sca-1^+^ and CD29^+^gp38^+^Sca-1^-^. Co-cultures established using the CD29^+^ subcultures A, B, and C, exhibited different hematopoietic support capacity, again reflective of the heterogeneity within this stromal fraction (Figure 3A). Replication experiments therefore involved independent sorts and growth of stromal fractions for 4 weeks. For reasons of low subset size it was not possible to perform replicate tests of hematopoiesis for any one sorted stromal fraction. Since the independently sorted and grown populations reflect distinct outgrowths of a heterogeneous population as seen for subcultures of the CD29^+^ fraction, it is not possible to determine with certainty a measure of the hematopoietic support capacity for impure stromal fractions. It was anticipated, however, that if a stromal fraction could be defined on the basis of multiple markers that it would be more likely to reflect a single stromal cell population.

The phenotype of cells produced in co-cultures was found to be similar across many stromal subsets analyzed across 11 different experiments (Figure 3B). Furthermore, after 21 days, there was a predominance of L-DC produced in most co-cultures with variable representation of myeloid cells and progenitors. Only 5G3 co-cultures showed evidence of a small subset of regulatory DC which are known to be transiently produced (Periasamy et al., 2013). Initial experiments confirmed no production of T or B lymphoid cells in any co-culture established with spleen stroma or the 5G3 cell line, consistent with previous reports (Periasamy et al., 2009; Petvises and O’Neill, 2014b), and also with early studies on longterm stromal cultures of spleen (O’Neill et al., 2004, 2014). Some stromal co-cultures showed more extreme changes in cell production, most notably subculture CD29^+^A which produced a high number of cells with a predominant population of progenitors (Figure 3B).

Relative to the input number of Lin^-^ bone marrow progenitors, 5G3 co-cultures yielded a 2.034 ± 0.350 (mean ± SE) (*n* = 9) increase in cell number after 21 days (Figure 3A). This fold change increase is more significant due to the fact that it represents cell production measured at 21 days, and does not take into account cells produced and lost at biweekly medium change. The question of whether stromal co-cultures cause expansion or maintenance of HSPC can be addressed as well. Nineteen of 20 co-cultures shown across 9 experiments in Figure 3 gave a yield of progenitors which was less than the input number of Lin^-^ bone marrow progenitors. However, CD29^+^A cultures gave a 2.7-fold increase in progenitors collected after 21 days compared with input cell number (Figure 3B). This initial study confirmed that spleen contains subsets of CD29^+^ cells which can support the expansion of HSPC cells. Despite the low overall yield of cells, gp38^+^ stromal co-cultures appear to maintain a higher proportion of progenitors.

### Stromal Cell Capacity to Maintain Hematopoietic Progenitors

The question of whether stromal co-cultures can maintain and expand hematopoietic stem and progenitor cells (HSPC) *in vitro* has clinical importance. We therefore attempted to identify HSC in several co-cultures established with freshly prepared splenic stroma. Non-adherent cells collected from co-cultures were stained to detect Lin^-^CD45.2^+^Sca-1^+^c-Kit^+^ HSPC. Only Lin^-^CD45.2^+^c-Kit^+^ cells could be detected in co-cultures established above the CD45^-^ and CD140a^+^ spleen stroma fractions tested, and none in 5G3 co-cultures (Figure 4A). These cells could reflect previously described common myeloid progenitors (Akashi et al., 2000), myeloid/dendritic progenitors (Fogg et al., 2006) or common dendritic progenitors (Liu et al., 2009). Previously we reported that HSPC appear to be tightly adhered to stroma so their isolation may not be easily achieved (Wilson et al., 2000). It is also possible that HSC may be present, but after for 3–4 weeks of culture, attain a different phenotype. In particular, culturing of Lin^-^Sca-1^+^c-Kit^+^ cells over stroma may lead to loss of the Sca-1 marker, a phenomenon reported previously for mesenchymal stem cells *in vitro* (Deng et al., 2015). Previously we reported evidence for maintenance of low numbers of HSPC in spleen longterm stromal cultures through ability to induce hematopoietic reconstitution in irradiated host mice (O’Neill et al., 2014).

We showed previously that the 5G3 stromal cell line can maintain bone marrow-derived HSC and MPP and support hematopoiesis to give a mixed population of L-DC and progenitors (Petvises and O’Neill, 2014b). Here we tested the capacity of several *ex vivo* spleen stromal fractions to support hematopoiesis in overlaid Lin^-^Sca-1^+^c-Kit^+^Flt3^-^CD150^+^ HSC. When non-adherent cells were collected and stained for marker expression, a substantial population of CD11b^+^CD11c^+^ cells was detected for each of the gp38^+^ and Thy1^+^ stromal fractions as well as for 5G3 (Figure 4B). These cells were further shown to express F4/80 but not MHC-II consistent with production of L-DC. A substantial CD11b^-^CD11c^-^ subset was detectable particularly for 5G3 cocultures (73%) while lower numbers of progenitors were present in co-cultures of gp38^+^ (18.9%) and Thy1^+^ (9.53%) stroma (Figure 4B). This subset is expected to contain hematopoietic progenitors.

### Fine Definition of Stromal Subsets in Spleen

**Figure 5:**
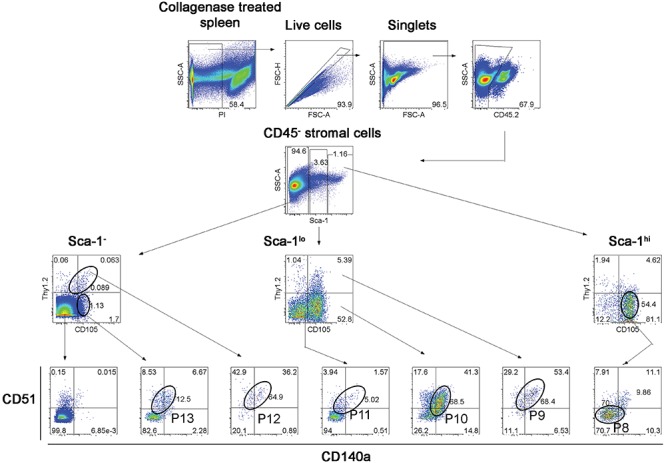
Flow cytometric analysis of stromal subsets in spleen. Stromal cells were isolated from murine spleen using collagenase treatment and stained with antibodies specific for CD45.2, Sca-1, Thy1.2, CD105, CD51 and CD140a. Prior to flow cytometry, cells were incubated with propidium iodide for gating live (PI^-^) cells using PI versus SSC analysis. Singlets were then selected on the basis of FSC-H and FSC-A staining. Cells were then gated on FSC to eliminate red blood cells. Multicolor analysis was then used to detect subsets amongst the CD45.2^-^ population of non-hematopoietic stromal cells. Fluorescence minus one (FMO) controls were used to set gates. Numbers in quadrants, or on circle gates, represent % specific binding. Distinct subsets were identified as P8, P9, P10, P11, P12, and P13. Data reflect 3 independent experiments.

Further experiments were therefore aimed at identification of subsets of more defined phenotype and hematopoietic support capacity. Splenic stroma was characterized using five cell surface markers to delineate subsets amongst the total *ex vivo* population. The panel of markers comprised Thy1.2, Sca-1, CD105, CD51, and CD140a, informed by the 5G3 phenotype and data obtained here on the growth and hematopoietic support function of stromal fractions (Table 3 and Figure 3). Stromal cells were prepared from 6 day old murine spleens using collagenase treatment and stained for identification of subsets. Live (PI^-^), non-hematopoietic (CD45.2^-^) stromal cells were first gated. Further analysis showed three distinct populations based on level of Sca-1 expression as Sca-1^hi^, Sca-1^lo^ and Sca-1^-^ cells (Figure 5). These were subsequently analyzed for Thy1.2, CD105, CD51 and CD140a expression. The Sca-1^hi^ subset contained a single population (P8), while the Sca-1^lo^ subset contained three populations (P9, P10, and P11) (Figure 5). The Sca-1^-^ subset contained two populations of interest (P12 and P13) (Figure 5). The proportional representation of these subsets amongst splenic culture was measured in three replicate experiments. P10 was the most predominant subset, although all were representative of <1% of stromal cells in 6 day old spleens (Table 4). P9, P10, and P11 reflect cells expressing marker profiles similar to 5G3. Stromal subsets P8, P9, P10, and P11 were sorted from 6 day old spleen for further study. Only P9 and P10 grew *in vitro* and produced confluent stromal monolayers within 28 days. These cells expressed a common Sca-1^+^gp38^+^CD29^+^Thy1.2^+^ phenotype, no longer expressing detectable levels of the CD105 or CD140a markers used to isolate the starting population (Table 4).

**Table 4:** Characterization of specific subsets amongst the spleen stromal fraction.

(A) Subset representation.
**Subset^a^**	**Designation^b^**	**% cells^c^**

Sca-1^hi^Thy1.2^-^CD105^+^CD51^lo^CD140a^-^	P8	0.17 ± 0.068
Sca-1^lo^Thy1.2^lo^CD105^+^CD51^+^CD140a^+^	P9	0.06 ± 0.021
Sca-1^lo^Thy1.2^-^CD105^+^CD51^lo^CD140a^lo^	P10	0.52 ± 0.190
Sca-1^lo^Thy1.2^-^CD105^-^CD51^+^CD140a^lo^	P11	0.04 ± 0.006
Sca-1^-^Thy1.2^lo^CD105^lo^CD51^+^CD140a^lo^	P12	0.03 ± 0.007
Sca-1^-^Thy1.2^-^CD105^+^CD51^+^CD140a^lo^	P13	0.06 ± 0.017

**(B) Phenotype of cells cultured out of stromal subsets.**

**Subset^b^**	**Cell number plated^d^**	**Phenotype of 28 day stroma^e^**

P8	(2.8 ± 0.5).10^4^ (*n* = 5)	No growth
P9	(7.6 ± 1.5).10^3^ (*n* = 5)	Sca-1^+^gp38^+^CD51^lo^CD105^-^CD29^+^ER-TR7^-^CD140a^-^Thy1.2^+^
P10	(9.8 ± 1.6).10^3^ (*n* = 5)	Sca-1^+^gp38^+^CD51^-^CD105^-^CD29^+^ER-TR7^-^CD140a^-^Thy1.2^+^
P11	(1.3 ± 9.6).10^2^ (*n* = 5)	No growth

^a^*The stromal the fraction of neonatal 6-day old spleen was isolated by collagenase digestion, stained for markers, and then delineated through flow cytometric gating of CD45^-^ cells.*^b^*Subset designation is shown in Figure 5.*^c^*Data reflect mean ± SE (n = 3).*^d^*Subsets were cultured for 28 days to determine capacity to form a monolayer.*^e^*Monolayers were trypsinised and cells stained with antibodies to determine phenotype flow cytometrically*.

Several stromal cell isolates including gp38^+^, CD140a^+^, CD105^+^, P9 and P10 were sorted for direct comparison with 5G3 in terms of hematopoietic support capacity. Ten independent experiments compared hematopoietic support capacity of a test stroma in relation to 5G3 used as the internal control (Table 5). Stromal cells were grown for 28 days to achieve minimal confluence and then stromal monolayers were overlaid with a constant number of Lin^-^ bone marrow progenitors. Non-adherent cells produced were removed weekly at medium change. Cells removed at 21 days were assessed in terms of yield and phenotype of cells produced. 5G3 showed reproducible capacity to support hematopoiesis with an average (±SE) yield relative to input cell number of 1.8.1 ± 0.18 across 10 experiments (Table 5). Capacity of test stroma to produce hematopoietic cells was then calculated relative to production in control co-cultures. The gp38^+^, CD140a^+^ and CD105^+^ stromal fractions gave yields less than that of 5G3 co-cultures, with CD105^+^ stroma being a very weak supporter. Two P9 co-cultures gave cell yields 2.71× and 3.76× greater than 5G3 (Table 5). Two P10 co-cultures gave distinctly different yields of 7.11× versus 0.280×, perhaps reflecting the outgrowth of variant stromal subsets as seen previously for CD29^+^ subpopulations (Figure 3). When cells produced in stromal co-cultures were assessed phenotypically, high numbers of L-DC and myeloid cells were represented with very few cDC-like cells. Only 5G3 produced cDC-like cells which were recently shown to resemble regulatory DC (Petvises et al., 2018). Each of the gp38^+^, CD140a^+^ and CD105^+^ stroma were high producers of L-DC relative to myeloid cells resembling 5G3, although their overall cell yield was lower than 5G3. Both P9 or P10 stroma produced even numbers of L-DC and myeloid cells. All co-cultures maintained a small population of CD11b^-^CD11c^-^ cells thought to contain myeloid progenitors and precursors (data not shown).

**Table 5:** *In vitro* hematopoietic support capacity of defined stromal subsets.

(A) Productivity of co-cultures established with stromal subsets.
**Experiment^a^**	**Test**	**Control**	**Relative yield in control^b^**	**Yield test/control^c^**

1	gp38+	5G3	1.80	0.178
2	gp38+	5G3	1.90	0.474
3	CD140a+	5G3	2.68	0.119
4	CD140a+	5G3	2.58	0.214
5	CD105+	5G3	1.80	0.044
6	CD105+	5G3	1.48	0.083
7	P10	5G3	1.90	**7.11**
8	P10	5G3	1.96	0.280
9	P9	5G3	1.48	**3.71**
10	P9	5G3	0.51	**2.75**
Mean ± SE			1.81 ± 0.18	

^a^*Each experiment involved two cocultures established by plating sorted test stroma and control stroma (5G3), with growth for 28 days to achieve near-confluent monolayers. P9 and P10 reflect subsets described in Figure 5. A constant number of Lin^-^ bone marrow cells (between 3.3 × 10^*5*^ and 7 × 10^*5*^) was then overlaid over stroma and cultured for 21 days with complete removal of non-adherent cells weekly. Cell yield at 21 days was calculated.*^b^*Cell production relative to input cell number was constant for 5G3 co-cultures.*^c^*Cell production in test versus control (5G3) co-cultures was calculated for each experiment. Values >1.0 are shown in bold*.

**Table:** 

(B) Proportion of myeloid subsets produced in stromal co-cultures.
	**% L-DC^a,b^**		**% cDC-like^a,b^**		**% Myeloid^a,b^**	
	**1st Experiment**	**2nd Experiment**	**1st Experiment**	**2nd Experiment**	**1st Experiment**	**2nd Experiment**

gp38+	81.0	86.9	0.14	0	18.9	13.1
CD140a+	75.1	65.6	0.08	0.03	24.8	34.3
CD105+	87.9	39.3	0	0.02	12.2	60.5
P9	58.5	47.8	0.03	0	41.2	52.6
P10	52.4	47.8	0.01	0.04	47.6	52.1
5G3^c^	90.5 ± 1.77		2.63 ± 1.42		7.02 ± 0.41	


*^*a*^See legend to table above. Data are shown for 2 independent experiments for test stroma.^*b*^Flow cytometry was used to identify myeloid cells produced in 21 day co-cultures through staining with CD11b, CD11c, MHC-II and F4/80. Data shown represent proportion of subset relative to total myeloid subset defined as CD11c^+^ and/or CD11b^+^ cells.^*c*^Data for 5G3 is shown as mean ± SE (n = 5)*.

## Discussion

Traditionally, the spleen is viewed as an immune organ which also supports erythropoiesis (Mebius and Kraal, 2005; Cesta, 2006). However, the spleen also plays an active role in extramedullary hematopoiesis during times of stress, infection and inflammation (Cesta, 2006; Johns and Christopher, 2012; Yamamoto et al., 2016). In this study, we have studied steady-state spleen to better understand the composition of stroma, its heterogeneity and growth capacity with a view to assessing its ability to support hematopoiesis. The hypothesis tested here is that spleen contains perivascular reticular cells of mesenchymal lineage which support hematopoiesis. Results confirm that the spleen stromal population is highly heterogeneous, but contains subsets which resemble mesenchymal perivascular cells which secrete CXCL12 and SCF and can support hematopoiesis. Clearly spleen contains a mixture of stromal cells of different phenotype which serves to emphasize the complexity of the stromal network and its important role in hematopoiesis and immune response development. Previously this lab showed grafting of splenic stromal cells sorted on the basis of a single marker (Tan and Watanabe, 2014, 2018). Those grafts showed the presence of hematopoietic cells and full development of splenic architecture.

Stromal subsets have been assessed for morphology, phenotype, growth and hematopoietic support capacity. Stromal fractions depleted of cells expressing CD29, gp38, CD105, Thy1.2 or CD140a failed to establish monolayers in culture, identifying these as important markers of cells which can grow *in vitro*. Stromal cells that grow out of 28-day cultures consistently show a Sca-1^+^gp38^+^Thy1.2^+^CD29^+^CD51^+^ phenotype with some variability in expression of CD105 and CD140a, indicative of mesenchymal lineage cells readily expanded *in vitro*. However, caution needs to be exercised in regard to marker expression, since culturing conditions can lead to changes in surface maker expression (Pevsner-Fischer et al., 2011).

The co-culture assay involving overlay of Lin^-^ bone marrow cells above 5G3 stroma has been extensively described (Periasamy et al., 2009; Periasamy and O’Neill, 2013; Petvises and O’Neill, 2014b). Due to the low subset size of stromal fractions, and the need to culture stroma for 4 weeks to obtain enough cells for analysis of hematopoietic support capacity, it was difficult to gain reproducible data on hematopoietic support capacity for all stromal fractions. For this reason, a number of complementary experiments were attempted to sort and compare subsets of different marker phenotype. Overall the data confirmed hematopoietic support capacity for subsets resembling 5G3 stroma, with production of L-DC, myeloid cells and very few or no cDC-like cells in co-cultures. Exceptions were one of the subcultures of CD29^+^ stroma, one P10 stromal culture and the two P9 cultures. Stromal co-cultures also maintained a small population of progenitor/precursor cells within the CD11b^-^CD11c^-^ subset.

Heterogeneity within splenic stromal subsets appears to contribute to variability in hematopoietic support capacity and this is very evident for P10 stroma. One out of 2 stromal cultures of sorted P10 cells was a high cell producer. Given the relatively large size of the P10 subset amongst the stromal subsets identified in Table 4 and Figure 5, it is possible that P10 is heterogeneous, and that further markers or single cell sequencing analysis will be needed to identify stroma which are strong supporters amongst this subset. In contrast, two isolates of the very small P9 subset were very strong supporters of hematopoiesis. Data shown here are consistent with a model whereby specific stromal cells, perhaps defined by the P9 and P10 subsets identified here, have superior hematopoietic support function.

The variability in hematopoietic support noted in this study parallels that seen amongst clonal cell line derivatives of the original parental STX3 splenic stromal line (Despars and O’Neill, 2006a; Periasamy et al., 2009). In that study, cloned stromal lines derived from STX3, including 2A8, 5G3, and 10C9, were good but variable supporters, whereas 3B5 was a much weaker supporter (Periasamy et al., 2009). In this study, the equivalent *in vivo* Sca-1^+^gp38^+^Thy1.2^+^CD29^+^CD51^+^ stromal cell subset has been shown to grow *in vitro* and to support hematopoiesis. However, since the markers which define this subset are not lineage-specific, heterogeneity may still exist within this rare population. Analyses of growth capacity have indicated the importance of CD140a and CD105 as markers defining stromal subsets which can grow *in vitro* and show hematopoietic support. However, these were also found to be elusive markers showing variable and sometimes weak expression, perhaps subject to *in vitro* growth conditions.

While the main focus of the paper has been on identification of stromal cells which reflect perivascular reticular cells akin to those described in bone marrow, we have also confirmed that some stromal fractions have capacity to expand hematopoietic progenitors. We have so far been unable to study these progenitors in detail due to the small number of cells which can be collected from stromal co-cultures. Future work will concentrate on the very small P9 and P10 fractions and their capacity to support hematopoiesis. Recent studies now describe splenic stromal cells which act as niches for HSC under conditions of extramedullary hematopoiesis (Inra et al., 2015; Oda et al., 2018). However, these reports do not directly identify stromal cells nor do they test their capacity for supporting hematopoiesis. They are concerned with the contribution of spleen to hematopoiesis under stress conditions, and do not consider a role for spleen in hematopoiesis under steady state conditions in normal animals. This study contributes information on the stromal cell type which signals hematopoiesis in the steady-state, as well the myeloid cell types produced. The contribution of spleen to hematopoiesis is an important consideration particularly in regard to aging and HSC transplantation in clinical settings.

## Ethics Statement

This study wascarried out in accordance with the recommendations of the Australian Code for Care and Use of Animals for Scientific Purposes, 8th Edition (2013). Work was conducted according to protocol # A2013/11 approved by the Animal Experimentation Ethics Committee of the Australian National University.

## Author Contributions

HL designed and performed experiments, analyzed and assembled the data, and wrote and reviewed the manuscript. HO designed the experiments, analyzed and interpreted the data, and wrote and reviewed the manuscript.

## Conflict of Interest Statement

The authors declare that the research was conducted in the absence of any commercial or financial relationships that could be construed as a potential conflict of interest.
