# Supplementary material for: Identification of Stromal Cells in Spleen Which Support Myelopoiesis
Source: Front Cell Dev Biol. 2019 Jan 24;7:1. doi: 10.3389/fcell.2019.00001 (PMC6354566; doi:10.3389/fcell.2019.00001)
Supplement: Supplementary file 1 [file Table_1.docx]

**Supplementary Table S1:**

**Growth assessment of CD29^+^ stromal subsets**

| **Cell fractions**$\text{‡}$**#** | **Growth**$\text{†}$ | **Phenotype of 28 day stroma^§^** | | | | | | | |
| --- | --- | --- | --- | --- | --- | --- | --- | --- | --- |
|  |  | Sca-1 | gp38 | CD51 | CD105 | CD29 | ERTR7 | CD140a | Thy1.2 |
| CD29^-^CD105^-^ | - |  |  |  |  |  |  |  |  |
| CD29^+^CD105^-^ | * | +++ | +++ | ++ | - | +++ | - | - | +++ |
| CD29^+^CD105^+^ | * | +++ | +++ | + | - | ++ | - | + | +++ |
| gp38^-^CD105^-^ | - |  |  |  |  |  |  |  |  |
| gp38^-^CD105^+^ | - |  |  |  |  |  |  |  |  |
| CD29^+^gp38^-^ | * | +++ | +++ | + | + | +++ | - | + | +++ |
| CD29^-^gp38^-^ | - |  |  |  |  |  |  |  |  |
| CD29^+^gp38^+^Sca-1^+^ | * | +++ | +++ | +++ | ++ | +++ | - | ++ | +++ |
| CD29^+^gp38^+^Sca-1^-^ | * | +++ | +++ | +++ | +++ | +++ | - | + | +++ |

$\text{‡}$ Stromal cells were isolated from murine spleen using collagenase treatment and stained with antibodies specific for CD45.2 to gate out hematopoietic cells, and either CD29, Sca-1gp38 or CD105 to gate stromal cells. Sorted cell subsets were cultured in sDMEM for 28 days.

# Number of cells plated was 5x10^4^ in 5 ml sDMEM.

$\text{†}$ Cell growth: confluent by 10 days (**), confluent by 28 days (*), no confluence (-).

§ Cells were trypsinised and stained with antibodies to determine phenotype.

+++, >80% cells expressing; ++, 50-80%; +, 10-50%; -, < 10%.
